# Multilevel associations of peer cognitive factors and adolescent cannabis use in a legal recreational cannabis region

**DOI:** 10.3389/fpsyt.2024.1477000

**Published:** 2024-11-19

**Authors:** Emily A. Kenyon, Manshu Yang, Tammy Chung, Anna C. Wilson, Sarah W. Feldstein Ewing

**Affiliations:** ^1^ Department of Psychology, College of Health Sciences, University of Rhode Island, Kingston, RI, United States; ^2^ Center for Population Behavioral Health, Rutgers the State University of New Jersey, New Brunswick, NJ, United States; ^3^ Department of Pediatrics, Oregon Health & Science University, Portland, OR, United States; ^4^ Departments of Psychiatry and Child Psychiatry, University of Connecticut School of Medicine, Farmington, CT, United States

**Keywords:** adolescent, cannabis, longitudinal multilevel analysis, peer influence, peer norms, recreational cannabis legalization, social cognition

## Abstract

**Background:**

Cannabis use can have unintended, harmful consequences for adolescents, a developmental group that struggles with heightened pressure to align with peer attitudes and behaviors. The role of social-cognitive factors in shifting cannabis use dynamics remains under explored, particularly in states where recreational cannabis use is legal.

**Objectives:**

The present study examined multilevel longitudinal associations between resistance to peer influence, peer norms, and adolescent cannabis use over the course of 12 months.

**Method:**

Participants were *N*=204 adolescents ages 15-19 (*M*_age_ = 18.68; 67% female) recruited via community outreach after the legalization of adult (age 21+) recreational cannabis use in the Portland, Oregon metropolitan region. Eligible participants endorsed 1+ heavy episodic drinking (HED) episode in the prior two months. Data were collected across four timepoints over 12 months. Multilevel latent growth curve modeling investigated associations between time-varying cognitive factors (resistance to peer influence, peer norms) and two cannabis outcomes (hazardous use, past-month use).

**Results:**

Findings showed individual increases in hazardous cannabis use over time were significantly associated with adolescents reporting higher peer norms (i.e., higher perceived prevalence and frequency of peer cannabis use) and lower resistance to peer influence. When assessing between-adolescent differences, hazardous cannabis use was only associated with peer norms. Individual variation over time and between-adolescent differences on past-month cannabis use was associated with peer norms, but not resistance to peer influence.

**Conclusions:**

Evolving cognitive factors like resistance to peer influence and peer norms may enhance understanding of longitudinal changes in hazardous cannabis use among adolescents and implicate helpful targets for prevention and intervention. It is a public health priority to identify factors that contribute to adolescent use trajectories in this period of growing cannabis legislation in order to guide the development of impactful prevention and intervention strategies.

## 1 Introduction

Recent national surveys estimate that 29% of 12^th^ grade U.S. adolescents report past-year cannabis use, with 20% using in the past 30 days, and 6.3% using near daily (1). Research demonstrating a direct public health effect of recreational cannabis legislation (RCL) on adolescent use is mixed (2, 3). A recent analysis across U.S. states found that both adolescents’ likelihood of endorsing cannabis use and use frequency were not related to RCL, whereas retail sales were associated with increased frequency among youth already using cannabis (4). Other studies suggest young people living in RCL states show increases in use post-legalization (5, 6). Recent evidence suggests that youth residing in RCL states have greater odds of transitioning to cannabis use (7) and past month use (8, 9) compared to youth in non-legalizing states. One key issue related to adolescent cannabis use is the steady decline in perceived risk that has mirrored increased medical and recreational legalization throughout the past decade (10–12). In addition, youth perceptions of cannabis as easier to access, which have also accompanied increases in cannabis legalization, have been observed to be associated with higher rates of frequent and heavy past-month use and transition into cannabis use disorder (CUD) in adulthood (13–15). Further, early initiation of cannabis (e.g., prior to age 14-15) increases risk of lifetime CUD diagnosis and poorer long term treatment response (16, 17).

Of particular concern, *hazardous* cannabis use is defined by use (e.g., the amount, frequency, and/or circumstances) that increases the risk for harm and health consequences (18). Hazardous use can increase accidents and injuries requiring emergency department visits (19), contribute to poor academic/occupational outcomes (20), and increase self-reported negative consequences in young adulthood (21). Adolescents have historically unmet treatment needs in addiction science, and clinical interventions for adolescent cannabis use often have underwhelming outcomes (22, 23).

Peer relationships play an important role in healthy adolescent development, and peer influence has a consistent and powerful impact on adolescent engagement in health risk behaviors such as cannabis use (24–26). Unsurprisingly, the proportion of substance using friends is among the strongest predictors of youth substance use initiation (27, 28) and research has shown a strong relationship between adolescents’ own use and their friends’ use (29, 30). Peer use has been directly related to both current and individual trajectories of cannabis use (31) and perceptions of close friends’ behavior (e.g., using, offering cannabis) has significantly predicted later cannabis use in longitudinal studies (32).

Social norms theory suggests that peers provide and reinforce *norms*—a group’s shared values, attitudes, and behaviors that are shaped by sociocultural context (33, 34). Further, norms are social-cognitive by nature and promote or discourage context-dependent health behaviors (35). The broader literature focuses on two different types of norms: 1) *descriptive*, or the perception of the number of others who partake in a behavior (i.e., prevalence and frequency), and 2) *injunctive*, related to the perceived approval of the behavior by others. Peer norms have demonstrated a robust relationship with individual cannabis use across a variety of adolescent studies (36–38). Higher cannabis peer norms may reflect higher perceived prevalence and frequency of their peers’ cannabis use, and/or perceived approval from peers about using cannabis.

Importantly, enhanced perceptions of peer use in this age group appear to be a highly relevant cognitive factor driving the initiation and escalation of cannabis use (39). Young people typically hold misperceptions of close peers’ cannabis and other substance use that exceeds rates of actual use or presume more substance related permissive attitudes among their peers (40, 41). Though there are robust associations between peer norms and cannabis use (42), the relationships between shifting cognitive factors, their association with cannabis use patterns, and how they may covary over time for adolescents residing in areas with RCLs have been under evaluated (43, 44).

### 1.1 Current study

The purpose of the present study was to examine between- and within-person associations for two social-cognitive factors (resistance to peer influence, peer norms) and adolescents’ cannabis use. Specifically, at the between-person level, we examined whether levels of average resistance to peer influence and peer norms were associated with average levels of cannabis use (hazardous use, past-month use) over the 12-month study period. We hypothesized that adolescents who reported higher peer norms (i.e., higher perceived prevalence and frequency of peer cannabis use) and lower resistance to peer influence would be more likely to report past-month cannabis use and more hazardous cannabis use.

We also examined the association between social-cognitive factors and cannabis use at the individual (i.e., within-person) level over time. We tested the relationships between resistance to peer influence and peer norms with both cannabis use outcomes. To inform more impactful prevention and intervention programming, this level of analysis provides unique information about contextual contributions to the association between peer factors and cannabis outcomes relative to an individual’s average level. We hypothesized a negative association between resistance to peer influence and cannabis use, such that adolescents would report lower resistance to peer influence if they also endorsed past month use and reported more hazardous cannabis use than usual. We also hypothesized a positive association between peer norms and cannabis use, such that adolescents would report higher peer norms if they also endorsed past month use and more hazardous cannabis use than usual.

## 2 Method

### 2.1 Participants and procedures

This study is a secondary data analysis of a NIH-funded translational study (1R01AA023658-01; PI Feldstein Ewing). Youth were recruited via community outreach between 2017 and 2018 in the Portland, Oregon metropolitan region. Notably, the study began after state legislation was passed in 2015 legalizing recreational cannabis use for people aged 21 and older, and data collection was completed in 2019 prior to the COVID-19 pandemic onset in March 2020.

All adolescents were engaged in alcohol use as inclusion criteria and were not seeking treatment. Eligible participants were 14-19 years old at baseline and currently engaged in heavy episodic drinking (HED; defined as >1 HED episode in the prior two-month period, defined as consuming >3 drinks for girls and >4 for boys per drinking occasion). Exclusion criteria followed translational requirements of the study: MRI contraindications (e.g., metal in body, pregnancy), left-handedness (due to differences in functional brain organization), history of brain injury/illness, loss of consciousness ≥ 2 minutes, current use of antipsychotics/anticonvulsants, neuro-developmental disorder, and >3 past month occasions of substances other than cannabis, alcohol, or nicotine (e.g., non-prescribed use of opioids, stimulants). To corroborate self-report, participants were breathalyzed to ensure BrAC=0 and provided a urine drug screen before all study visits.

Parent informed consent/adolescent assent was obtained for youth under age 18 while those age 18+ provided independent informed consent. Once enrolled, participants were randomized to one of two brief individual alcohol interventions (two sessions, one hour each, one week apart). Given that effect sizes in the target behavior (alcohol reductions) were small and differences between treatment conditions minimal (45, 46), intervention assignment was not accounted for in the present study.

Participants were asked to complete surveys at four timepoints: baseline, 3-, 6-, and 12-months and received up to $250 for completing all study components. Due to the parent study design, only those who completed a full baseline visit were followed at 3 months (85%). Among these participants, 96% were retained at the 6- and 12-month timepoints. All study procedures were approved by the participating institutional review board.

### 2.2 Measures

#### 2.2.1 Demographics

At baseline a series of questions assessed participant age, sex assigned at birth, race and ethnicity, highest grade completed, and substance use history.

#### 2.2.2 Hazardous cannabis use

The Cannabis Problems Questionnaire for Adolescents (CPQ-A) is an empirically supported and widely used 27-item measure (47) to assess hazardous cannabis use in this developmental age group at each study timepoint. This measure is designed to capture a wide range of consequences associated with cannabis use for adolescents (e.g., worrying about the amount of money spent on cannabis). All items are answered yes/no and summed to a total score; baseline internal consistency of α **= .**87 showed good reliability in this sample.

#### 2.2.3 Past-month cannabis use

This was assessed using the Timeline Follow-Back (TLFB) (48), an interviewer-based calendar measure that generates the number of prior-month days that cannabis was used, which allowed us to calculate the proportion of days used for this study (*M*_T1_ **=** 0.24, *SD*=0.33, *Med*_T1_=.07, range 0-1.00) at each timepoint. This reflects that the average participant used cannabis on approximately one-quarter of prior-month days assessed at baseline. Due to a substantial number of participants reporting zero past-month use days (33.5%), as well as others reporting daily or near-daily use (>20 days; 16%), the variable was dichotomized (yes/no) across all timepoints to indicate whether the participant used cannabis in the prior month and was treated as a binary outcome in the models.

#### 2.2.4 Resistance to peer influence

The Resistance to Peer Influence Scale (RPI) (49) asks participants to choose between 10 pairs of statements by deciding which person they are most like (e.g., “Some people take more risks when they are with their friends than they do when they are alone” vs. “Other people act just as risky when they are alone as when they are with their friends”) and rating the best descriptor as “Really True” or “Sort of True” for them. Responses are coded on a 4-point scale from 1 (“Really True” for descriptor A) to 4 (“Really True” for descriptor B). After three items are reverse coded, the total scale score is calculated by summing all items and computing an average (range 1-4) with higher scores reflecting greater resistance to peer influence. Previous research indicates that RPI increases most significantly between ages 14-18 and stabilizes in emerging adulthood (49). The scale demonstrated adequate reliability with a baseline internal consistency of α = .73 in this sample.

#### 2.2.5 Peer norms

Peer norms were measured with four items to assess the perceived prevalence and frequency of cannabis use (descriptive norms), such as 1) whether most friends use cannabis; 2) how often their friends use cannabis; 3) how often most of their friends get intoxicated when using cannabis; and 4) whether most people their age use cannabis (50). The first item was measured on a yes/no scale, the two items assessing frequency were measured on a 5-point scale from “never” to “always,” and the fourth item was measured on a 4-point scale from “disagree a lot” to “agree a lot.” Items were standardized with a Z transformation before being summed and averaged to calculate a mean score, where higher scores indicate higher peer norms. The measure demonstrated good reliability with baseline internal consistency of α = .79. The original items used the term “marijuana” instead of the scientifically preferred “cannabis” as we now refer to it throughout (44).

### 2.3 Analytic strategy

We utilized multilevel latent growth curve modeling to explore the overall trajectories of change and associations over time, as well as the individual differences in the magnitudes of these associations (51, 52). Models were fit by specifying the between- and within-person levels of the longitudinal data to account for the complex clustering due to the repeated-measures design.

Two multilevel models were tested for each cannabis outcome (past-month and hazardous use). Each model utilized a multilevel structural equation model (SEM) approach that included two random effects for growth (i.e., random intercept and slope), and two random effects that captured the effects of RPI and peer norms on predicting cannabis outcomes across the study period (baseline, 3-, 6-, and 12-months). We utilized the multilevel latent variable modeling framework in Mplus (53) to facilitate the inclusion of time-varying covariates, and to disaggregate the within-person and between person effects.

We examined the between-person effects to test whether, on average, individuals reporting higher perceived prevalence and frequency of cannabis use among peers (i.e., higher peer norms) and lower RPI are more likely to endorse past-month and hazardous cannabis use. The random intercepts of cannabis outcomes (i.e., person-level means) were regressed on the random intercepts (i.e., person-level means) of each peer predictor variable.

To test the hypothesized within-person effects, models examined the individual associations of each time-varying peer predictor with past-month and hazardous cannabis use over time. A latent person-mean centering approach was applied to the peer norm and RPI variables to facilitate the interpretation of within- and between-person effects (54). Thus, an adolescent’s score for a given timepoint is relative to their usual or average hazardous cannabis use level.

Time was mean centered to reflect months from the mid time point (i.e., -5.25, -2.25, 0.75, and 6.75) and was a predictor added in each model to control for (i.e., detrend) any effect of time on the cannabis outcomes (55). Bayesian estimation with Markov Chain Monte Carlo (MCMC) simulation was used with non-informative default priors in Mplus under the missing at random (MAR) assumption (56). Data management was conducted using IBM SPSS v27, and analyses were conducted using SAS Studio v3.8 (57) and Mplus v8.6 (53).

## 3 Results

### 3.1 Preliminary analyses

The full sample comprised *N*=204 adolescents ranging in age from 15-19 (*M*=18.68); 66.7% identified their sex assigned at birth as female, and reflecting the region from which this sample was recruited, the majority reported their race as White (75.5%). Two-thirds reported past-month cannabis use at baseline (*n*=131, 66.5%). Among those reporting lifetime cannabis use (*n*=162, 79.4%), the average age of first cannabis use occurred between 15-16 years of age (*M*=15.4). Most participants (88.8%) reported past-month alcohol use and an average four past-month drinking days. Additional sample characteristics are provided in **Table 1**. Descriptive statistics among study variables are presented in **Table 2**.

**Table 1:** Descriptive characteristics of the baseline study sample (*N*=204).

	*M (SD)*	*n* (%)
*Age*	18.7 (1.0)	
*Age of First Cannabis Use* (*n*=162)	15.4 (2.0)	
*Past-Month Alcohol Use*		175 (88.8)
Total Drinking Days	4.0 (3.6)	
Drinks per Drinking Day	3.4 (2.3)	
*Assigned Sex at Birth*		
Female		136 (66.7)
Male		68 (33.3)
*Race*		
White		154 (75.5)
Black/African American		17 (8.3)
Native Hawaiian/Pacific Islander		7 (3.4)
Asian		36 (17.7)
American Indian/Alaskan Native		9 (4.4)
Hispanic/Latino		31 (15.2)
Other		5 (2.5)
*Ethnicity*		
Hispanic/Latino		34 (16.7)
Not Hispanic/Latino		170 (83.3)
*Highest Grade Completed*		
Less than 12^th^ grade		55 (27.0)
Graduated high school/GED		57 (27.9)
Some college		89 (43.6)
Technical/Associate degree		3 (1.5)

Age of first cannabis use was only assessed for those who reported lifetime cannabis use. For baseline past-month alcohol use, 7 reports are missing. Participants could endorse more than one option for the race category.

**Table 2:** Descriptive statistics of primary study variables across follow-up.

	*n* (%)	*Mean* (*SD*)		
*Timepoint*	*Past-month use*	*Hazardous cannabis use*	*Peer norms (Z score)*	*Resistance to peer influence*
Baseline (T1)	131 (66.5)	5.61 (4.99)	0 (.78)	2.96 (.50)
3-mo (T2)	98 (59.0)	4.81 (4.41)	0 (.75)	3.04 (.44)
6-mo (T3)	101 (62.7)	4.48 (4.05)	0 (.73)	2.98 (.47)
12-mo (T4)	90 (56.6)	3.98 (4.31)	0 (.74)	3.06 (.46)

Past-month cannabis use is dichotomized, percent of “yes” shown here.

Hazardous cannabis use was assessed with the Cannabis Problems Questionnaire for Adolescents (CPQ-A); possible range 0-27 (observed 0-22). Peer norms observed range -2.52-1.10. Resistance to Peer Influence (RPI); possible range 1-4 (observed 1.5-4).

For past-month use, the number of reports missing at each timepoint are: 7 (T1), 48 (T2), 43 (T3), 45 (T4).

For CPQ-A, the number of reports missing at each timepoint are: 40 (T1), 88 (T2), 67 (T3), 64 (T4).

For RPI and Norms, the number of reports missing are: 1 (T1), 36 (T2), 42 (T3), 39 (T4).

### 3.2 Primary analyses

**Figure 1:**
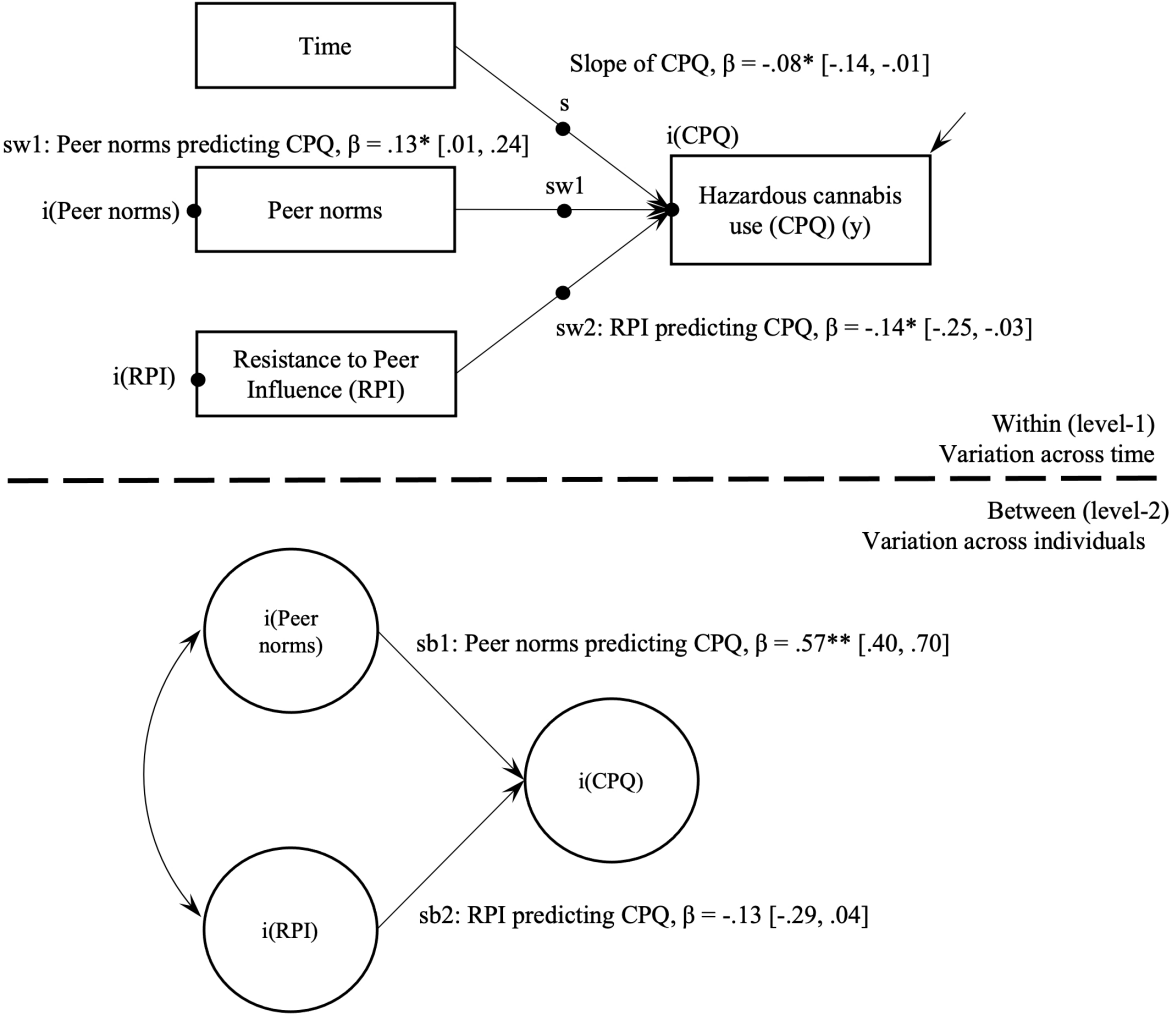
Multilevel path model of hazardous cannabis use and peer cognitive factors. CPQ, Cannabis Problems Questionnaire; RPI, Resistance to Peer Influence. *p < .05. **p < .001.

**Figure 2:**
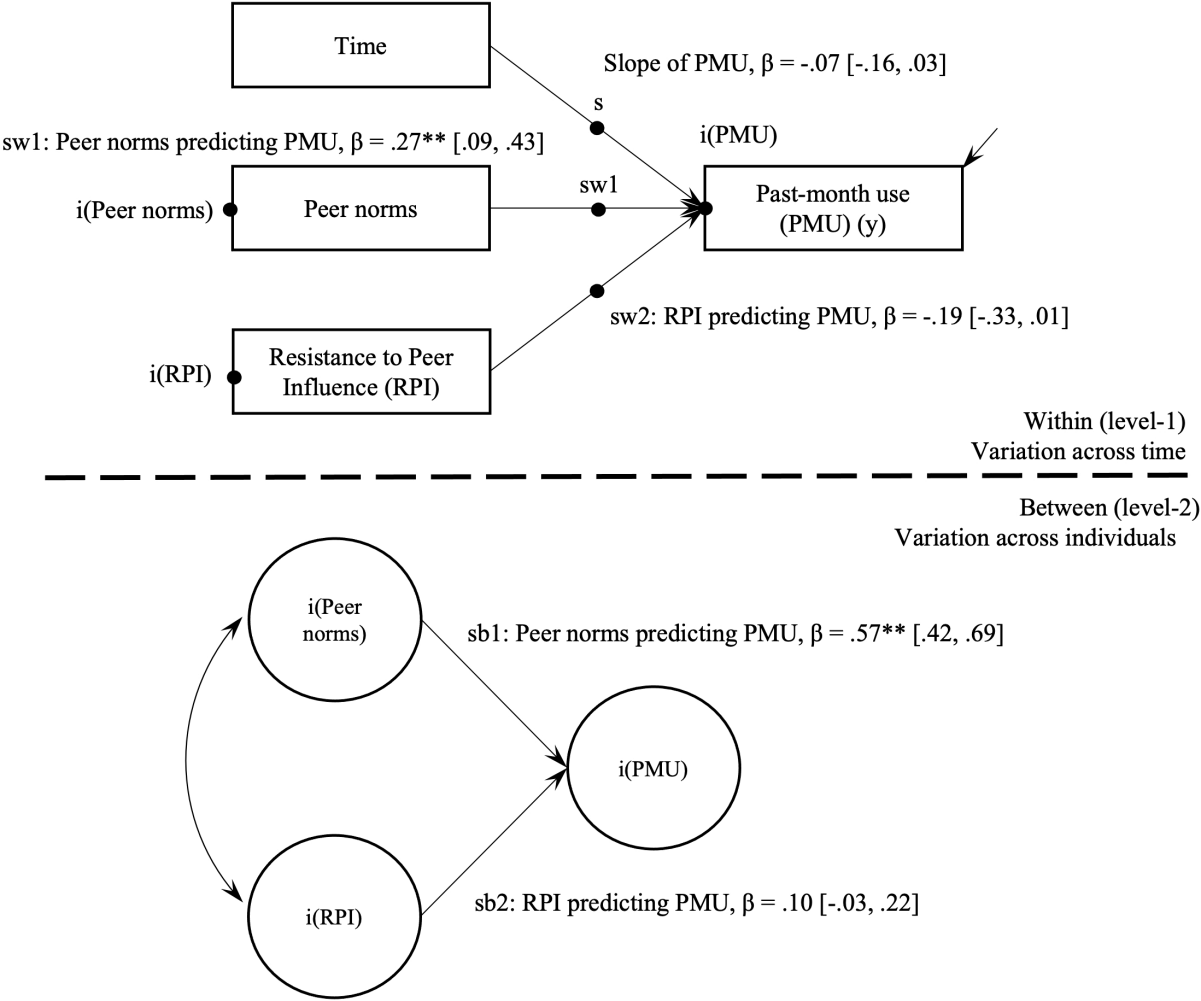
Multilevel path model of past-month use (yes/no) and peer cognitive factors. PMU, Past-month use; RPI, Resistance to Peer Influence. **p < .001.

Multilevel latent growth models evaluated the within-person and between-person effects of peer norms and RPI on hazardous cannabis use (Model 1; **Table 3**) and past-month use (Model 2; **Table 4**). **Figures 1**, **2** provide visual representations of these associations as two-level path models.

**Table 3:** Multilevel model results for hazardous cannabis use.

*Model 1*				
	*β*	*Posterior SD*	*95% CI*	*p-value*
*Within-Level Standardized Estimates (Averaged Over Clusters)*				
s | Slope (Hazardous use)	-.08	.03	[-.14, -.01]	.02
sw1 | Hazardous use on Norms	.13	.06	[.01, .24]	.03
sw2 | Hazardous use on RPI	-.14	.06	[-.25, -.03]	.02
Residual Variance (Hazardous use)	.67	.05	[.58, .76]	<.001
*Between-Level*				
sb1 | Hazardous use on Norms	.57	.08	[.40, .70]	<.001
sb2 | Hazardous use on RPI	-.13	.09	[-.29, .04]	.14
Intercept (Hazardous use)	2.21	.68	[ .87, 3.53]	<.001
Residual Variance (Hazardous use)	.65	.09	[.48, .82]	<.001

**Table 4:** Multilevel model results for past-month cannabis use.

*Model 2*				
	*β*	*Posterior SD*	*95% CI*	*p-value*
*Within-Level Standardized Estimates (Averaged Over Clusters)*				
s | Slope (Past-month use)	-.07	.05	[-.16, .03]	.18
sw1 | Past-month use on Norms	.27	.09	[.09, .43]	<.001
sw2 | Past-month use on RPI	-.19	.08	[-.33, .01]	.06
*Between-Level*				
sb1 | Past-month use on Norms	.57	.07	[.42, .69]	<.001
sb2 | Past-month use on RPI	.10	.07	[-.03, .22]	.13
Residual Variance (Past-month use)	.67	.08	[.52, .82]	<.001

Within-level residual variance is not directly estimated in this model with a binary dependent variable.

Model 1 indicated that hazardous cannabis use significantly declined over the study period (*β* = -.08, *95% CI* = [-.14, -.01], *p*=.02). In Model 2, endorsing past-month cannabis use did not significantly change over the study period, with the non-significant slope term (*β* = -.07, *95% CI* = [-.16, .03], *p*=.18) suggesting that on average adolescents did not move between the binary past-month use or non-use categories in a consistent direction during the 12-month period.

#### 3.2.1 Between-person associations

*Model 1.* On average, adolescents with higher hazardous cannabis use scores endorsed higher peer norms (*β* = .57, *95% CI* = [.40, .70], *p*<.001). Average level of RPI was not associated with hazardous cannabis use (*p*=.14).

*Model 2.* On average, adolescents who reported past-month cannabis use endorsed higher peer norms (*β* = .57, *95% CI* = [.42, .69], *p*<.001). Results showed a non-significant association between average levels of RPI and past-month cannabis use (*p*=.13).

#### 3.2.2 Within-person associations

*Model 1.* The hypothesized individual variability in hazardous cannabis use and associations with time-varying peer factors was supported. When adolescent-reported peer norms were higher than usual, their hazardous cannabis use also tended to be higher than usual (*β* = .13, *95% CI* = [.01, .24], *p*<.05). In the hypothesized negative direction, when an adolescent’s score on RPI was lower than usual, hazardous cannabis use tended to be higher than usual (*β* = -.14, *95% CI* = [-.25, -.03], *p*<.05).

*Model 2*. The hypotheses for individual variation in endorsement of past-month use and time-varying peer factors were partially supported. When peer norms were higher than usual, adolescents tended to endorse past-month cannabis use (*β* = .27, *95% CI* = [.09, .43], *p*<.01). Resistance to peer influence and past-month use showed a non-significant association (*β* = -.19, *95% CI* = [-.33, .01], *p*=.06).

## 4 Discussion

The present study investigated longitudinal associations between social-cognitive factors and adolescent cannabis use across a 12-month period. The repeated measures design elucidated how adolescent perceptions of their evolving peer landscapes impact individual variation in cannabis use over time. In line with previous literature demonstrating the salience of adolescents’ peer context on cannabis use, these outcomes support associations with peer norms and resistance to peer influence. Given the rapidly increasing recreational cannabis legalization landscape, it is a public health priority to identify factors that contribute to adolescent use trajectories in order to guide the development of impactful prevention and intervention strategies.

Assessing the weight of peer influence and youth efforts to maintain their ever-precarious social standing by behaving consistently with peer norms enhances our ability to prevent and intervene on hazardous adolescent substance use in a manner that is sensitive and responsive to this developmental stage (58, 59). For example, most adolescents report overwhelmingly positive social experiences resulting from substance use, as the emphasis may be on experimentation and peer relationships instead of potential consequences or downstream health risk (60). Additionally, by the time adolescents receive treatment for cannabis-related problems, many are immersed in cannabis-using friend groups due to self-selection and socialization processes (24). Youth may experience less motivation and greater perceived difficulty changing affiliations with cannabis-using friends in order to sustain abstinence goals following participation in substance treatment (61). It is important to understand how in response to increasing medical and recreational legalization, easier availability of cannabis and lowered perception of harm may relate to misconceptions about friend and peer behavior. Additionally, it is crucial to examine how these social-cognitive factors affect usage patterns and relationships with cannabis among this age group, particularly in the age of widespread social media use.

The perceived ability to resist peer influence has implications for cannabis use patterns due to the overwhelmingly commonplace nature of adolescent use taking place in primarily peer contexts. Perhaps reflecting fewer opportunities for adolescents to spend time in cannabis-using peer networks during the pandemic, the prevalence of 12^th^ grade past-year use showed the steepest decline ever documented by the Monitoring the Future study, dropping from 35% in 2020 to 31% in 2021 (1). As hypothesized, our results found a small, significant within-person effect for resistance to peer influence (RPI) and hazardous cannabis use, but, contrary to prediction, not significant between-person effects. In other words, when adolescents reported lower-than-their-average RPI at a given timepoint, they also reported higher hazardous cannabis use, but the hypothesis that adolescents who reported lower RPI in general would report more hazardous use was not supported. Acknowledging that youth naturally vary in their ability to resist peer influence over time helps to strengthen our understanding of contextual risk around hazardous cannabis use and related consequences. Findings underscore the need to improve prevention and intervention programming that target the period when modifying adolescent behavior could be most impactful, such as when they report lower-than-usual perceived ability to resist peer influence. Advancements in digital and smartphone-delivered technologies have facilitated the exploration of just-in-time adaptive interventions (JITAIs) (62) to tailor how harmful cannabis use can be reduced in the moment (63, 64). From a public health perspective, providing universal substance use screening and brief interventions in opportunistic settings (e.g., pediatric primary care visits, school-based health centers) before youth require treatment for harmful cannabis use is an important element of effective prevention. For adolescents who find themselves in peer contexts that promote use, enhancing refusal self-efficacy skills (e.g., ability to refuse/resist using cannabis) (65) and protective behavioral strategies (66) are promising directions to delay onset of cannabis use or reduce related harms, respectively.

Consistent with hypotheses, results showed significant positive associations between hazardous cannabis use and peer norms related to cannabis across within- and between-person levels. The large between-person effect size suggests that adolescents who reported higher peer norms on average also reported more hazardous cannabis use, which aligns with previous research (36–38, 67). The small within-person effect size reflects that adolescents tend to report higher peer norms when they also endorse more hazardous cannabis use compared to their usual or average level. This pattern held for the association between endorsing past-month use and norms, demonstrating a medium within-person effect and large between-person effect. This relationship may reflect that youth who use cannabis have an accurate sense of friends’ use patterns, thereby narrowing the gap between peers’ perceived and adolescents’ actual use. Exposure to cannabis-related content via peers on social media—a crucial emerging area of research—also shapes injunctive norms and is associated with adolescents’ cannabis use behaviors (67). Future research that follows adolescents and peers in their natural networks over time can clarify the direction of causal effects to identify intervention targets. Further exploration of additional social-cognitive factors (e.g., beliefs about long-term impacts of use, how peer use is viewed in terms of positive or negative expectancies) may also inform intervention.

Posing serious threats to public health, new high potency products and modes of delivery have accompanied burgeoning medical and recreational cannabis markets in the U.S (68, 69)., such as concentrates for inhalation with “vaping” devices and orally ingested “edibles” that are less detectable than smoking combustible cannabis flower (70, 71). Among 12^th^-grade youth engaged in cannabis use, the rate of past year cannabis vaping increased from 21.6% to 34.5% between 2017 and 2018 (72) and frequent past-month cannabis vaping has increased across all youth demographic groups (73). Future research should aim to better understand how norms relate to emerging patterns of cannabis use that can confer different actual and perceived risk of harm, as novel products being used or endorsed by peers (e.g., combustible vs. vaporized, edible types) continue to shape norms about cannabis use acceptability (74, 75). Namely, clinical efforts should tailor educational messages to account for perceptions of socially mediated and cannabis product-related risks and benefits.

### 4.1 Strengths and limitations

The current study had many strengths, including repeated-measures of adolescent cannabis use over four timepoints (baseline, 3-, 6-, and 12-months) with high participant retention. The parent study was designed to capture outcomes over long term follow-up, and data were collected prior to the COVID-19 pandemic. Recruitment took place in a major metropolitan area after adult medical and recreational cannabis legislation had been well-established, allowing us to study adolescent associations in the context of permissive RCL.

This study examined a sample of adolescents who endorsed alcohol use in the prior two months. Due to issues of power, we were unable to explicitly include alcohol or demographic variables such as age and education in analyses presented here. An important next step will be to consider patterns and trajectories of hazardous alcohol and cannabis co-use. The parent study was also not specifically designed to assess a cause-effect relationship of RCL on adolescent cannabis use or address primary questions about peer influences on use patterns. Given that policy and cultural context influence social-cognitive factors (e.g., attitudes, norms) bidirectionally, this sample may endorse more permissive peer norms than would be observed in other U.S. regions with stronger cannabis restrictions or with adolescent samples who do not drink alcohol. Other adolescent groups may report more hazardous levels of cannabis use, which may impact generalizability of these findings. Future directions include replicating findings with adolescents in states with and without RCL to understand how socioecological factors (e.g., policy) shape longitudinal relationships between social cognition and cannabis use.

## 5 Conclusion

The results from the current study provide an important contribution to the adolescent substance use literature by examining longitudinal within- and between-person associations among cannabis use and social-cognitive factors (resistance to peer influence, peer norms) in a U.S. region with legal recreational cannabis. The hypotheses were partially supported, such that within-person increases in hazardous cannabis use (i.e., endorsing more cannabis-related problems) were associated with lower resistance to peer influence (RPI) and higher perceived prevalence and frequency of cannabis use among peers (i.e., higher peer norms). At the between-person level, hazardous use was only associated with higher peer norms, and not RPI. As hypothesized, endorsing past-month cannabis use was associated with higher peer norms, whereas past-month use was not associated with lower RPI across within- and between-person levels. Taken together, study findings suggest that evolving social-cognitive factors like resistance to peer influence and peer norms around cannabis may be useful for understanding longitudinal changes in hazardous cannabis use among adolescents and implicate helpful targets for prevention and intervention.

## Data Availability

The datasets presented in this article are not readily available due to concerns regarding participant/patient anonymity. Requests to access the anonymised participant data should be directed to the corresponding author.
